# Enzyme assisted juice extraction from Dacryodes macrophylla as a potential bio-resource for wine production

**DOI:** 10.1016/j.heliyon.2023.e16443

**Published:** 2023-05-25

**Authors:** Aneh Phillins Aneh, Pride Ndasi Ngwasiri, Wilson Agwanande Ambindei, Makebe Calister Wingang, Ngwa Martin Ngwabie, Martin Benoit Ngassoum

**Affiliations:** a Department of Nutrition, Food and Bioresource Technology, College of Technology, The University of Bamenda, P.O. Box 39, Bambili-Bamenda, Cameroon; b Department of Process Engineering, ENSAI, University of Ngaoundere, P.O. Box 455, Ngaoundere, Cameroon; c Department of Chemical and Biological EngineeringNational Higher Polytechnic Institute, The University of Bamenda, P.O. Box 39, Bambili-Bamenda, Cameroon; d Department of Agriculture and Environmental Engineering, College of Technology, The University of Bamenda, P.O. Box 39, Bambili-Bamenda, Cameroon; e Department of Applied Chemistry, ENSAI, University of Ngaoundere, P.O. Box 455, Ngaoundere, Cameroon

**Keywords:** Bioresource, *Dacryodes macrophylla*, Juice, Extraction, Enzymes, Pectinase, Optimization, Wine

## Abstract

Atom fruit (*Dacryodes macrophylla*) is a Non-timber Forest Product (NTFP) that comprises a large seed, thick pulp, and a thin hard outer covering. The structural component of its cell wall and thick pulp make it difficult in extracting the juice. Also, *Dacryodes macrophylla* fruit is greatly underutilized, therefore the need to process and transform it into other value-added products. This work aims to enzymatically extract juice from *Dacryodes macrophylla* fruit with the aid of pectinase, ferment and test the acceptability of wine produced from this extract. The enzyme and non-enzyme treatments were carried out under the same conditions and their physicochemical properties such as pH, juice yield, total soluble solids, and Vitamin C were compared. A central composite design was used for the optimization of the processing factors for the enzyme extraction process. Enzyme treatment had a great impact on the juice yield (%) and Total soluble solids (TSS) (^0^Brix) of samples as it was as high as 81 ± 0.7% and 10.6 ± 0.02 ^0^Brix whereas, that of the non-enzyme treatments were 46 ± 0.7% and 9.5 ± 0.02 ^0^Brix respectively. However, the Vitamin C content of enzyme-treated juice decreased to 11.32 ± 0.13 mg/ml as compared to that of the non-enzyme-treated juice sample (15.7 ± 0.04 mg/ml). The optimum processing condition in the extraction of juice from the atom fruit was 1.84% enzyme concentration, 49.02 ֯C Incubation temperature, and 43.58 min Incubation time. During wine processing within 14 days of primary fermentation, the pH of the must decreased from 3.42 ± 0.07 to 3.26 ± 0.07 whereas the Titratable acidity (TA) increased from 0.16 ± 0.05 to 0.51 ± 0.0. The wine produced from *Dacryodes macrophylla* fruit showed promising results as its sensorial scores for all attributes including color, clarity, flavor, mouthfeel, alcoholic burn after taste and overall acceptability were all above 5. Thus, enzymes can be used to improve the juice yield of *Dacryodes macrophylla* fruit and hence, can be a potential bioresource for wine production.

## 1 Introduction

Fruits are among the most important foods of mankind as they are not only nutritive but are also indispensable for the maintenance of health. Fruits in fresh and processed forms not only improve the quality of our diet but provide essential nutrients like vitamins, minerals, and carbohydrates. Among fruit, grapes have seen the most technical and commercial use as substrates for winemaking. Today, a growing number of other fruits with different origins, cultivars, shapes, colors, tastes, and nutritive values, are gaining interest wine production [1]. Forest fruits are known for their medicinal benefits as both the fruits and seeds are beneficial [2]. *Dacryodes macrophylla* (Oliv.) Lam. is a non-forest timber fruit whose pulp contains juice [3], presumable rich in sugar, iron [4] flavonoids, *anti*-axidants and other phenolic compounds etc which stands as a potential unexploited good alternative fruits for wine production. Post-harvest losses of fresh fruits which most often is as a result of their high moisture content making them very perishable is one of the serious problems of these fruits [5]. The main challenge is the extraction of the sugar and other soluble solids from the pulp of *Dacryodes macrophylla* for the juice production and transformation into a shelf-stable product as wines.

Wine is an alcoholic beverage produced by the fermentation of sugars in the juice of any fruit usually grapes [5]. Fermented beverages have been known to mankind from time immemorial. A typical wine contains ethyl alcohol, sugar, acids, higher alcohols, tannins, aldehydes, esters, amino acids, minerals, vitamins, minor constituents like flavorings compounds, etc [6]. The consumption of wine has several health benefits like lowering mortality from cardiovascular disease and cancer, delaying dementia, and preventing arthritis [7]. Fruit wines are un-distilled alcoholic beverages that are made from grapes or other fruits such as bananas, mangoes, peaches, etc which are nutritive, tastier, and mild stimulants.

Fruit wines (non-grape wines) are gaining popularity among wine consumers because of their low alcohol content, unique aroma bouquet, organoleptic properties and additional health benefits [8]. The low level of domestication of grapes, the fruit of choice for wine production in the tropics has encouraged the search for alternative fruits such as bananas, mangoes, peaches, and apples for wine production in Cameroon and other tropical countries [9]. Over the recent years in different parts of the world, fruit wines have been prepared from several tropical fruits, such as banana [9], cagaita [10] mango [11], jackfruit [12], pineapple [13] in different parts of the world. The major problems associated with the use of tropical fruits in wine production include their low sugar content, high acidity, and the presence of an array of microorganisms other than wine yeast (*Saccharomyces cerevisiae* var ellipsoideus). In addition, tropical red wines have not been produced from tropical fruits because of their low content of extractable red pigments, unlike those in red varietal grapes [14].

Amongst this fruit, we also have *Dacryodes macrophylla* (Oliv.) Lam. Synonymously called *Canarium macrophyllum* Oliv. Or *Pachylobus macrophyllus* (Oliv.) Engl. They are Non-Timber Forest Products (NTFP) found in Equatorial Africa, in countries such as Cameroon, Equatorial Guinea, and Gabon [15]. The only part of *Dacryodes macrophylla* fruit whose use is known is the pulp. This pulp can be consumed directly or used in the making of juices [3]. Although not much analysis of the juice has been carried out, it is presumed that they are rich in sugar and the red color of the juice of *D. macrophylla* is presumed to indicate a high concentration of iron, which could be useful in treating anemia [4]. This thus makes it a potential bioresource for wine production. However, the fruit has a hard pericarp and large seeds causing it to have a thin pulp, thus rendering juice extraction through conventional methods as pressing difficult and resulting in a low juice yield [4]. There is the need to apply alternative extraction methods that facilitate the juice extraction process as Enzyme assisted extraction.

As reported by Refs. [16,17], enzyme-assisted juice extraction helps break down plant cell walls such as pectin, therefore, releasing juices trapped within them, helps prevent oxidation thus maintaining the color of the juice and improves also on its clarity. This thus results in greater juice yield and better color and clarity than conventional extraction methods. Therefore, can enzyme assisted extraction of juice from *Dacryodes macrophylla* results in optimal juice production, sugar extraction and with good characteristic for wine production*.* This study was therefore aimed at using enzymes to assist the juice extraction from *Dacryodes macrophylla* whose juice could serve as a potential bioresource for wine production.

## 2 Materials and methods

*Dacryodes macrophylla* Lam. fruits were purchased from marche Ndokoti Yaoundé. Pectinase enzyme (a mixture of pectinase, water and glycerine) and red wine yeast (*Saccharomyces cerevisiae*) were bought from VINO FERM (products by Brouwland Korspelsesteenweg Beverlo-Belgium). Sugar was purchased from local market and Potassium meta-bisulfite from LabTech all found at the Main market Bamenda Cameroon.

### 2.1 *Dacryodes macrophylla* juice extraction

The *Dacryodes macrophylla* fruits were manually plucked from stems and sorted to remove leftover leaves or stems from the fruit. Water at 40 ֯C was then used to ease washing and removal of the gummy substance found on the sorted fruits.

The washed fruits were then strained with a colander, weighed, and manually hand macerated with the help of a pistil. The control sample (untreated with enzyme) was prepared by measuring the volume of macerated fruit and then filtered through a muslin cloth. The clear juice extract free of seeds and skin was gotten.

### 2.2 Enzymatic treatment

**Fig. 1:**
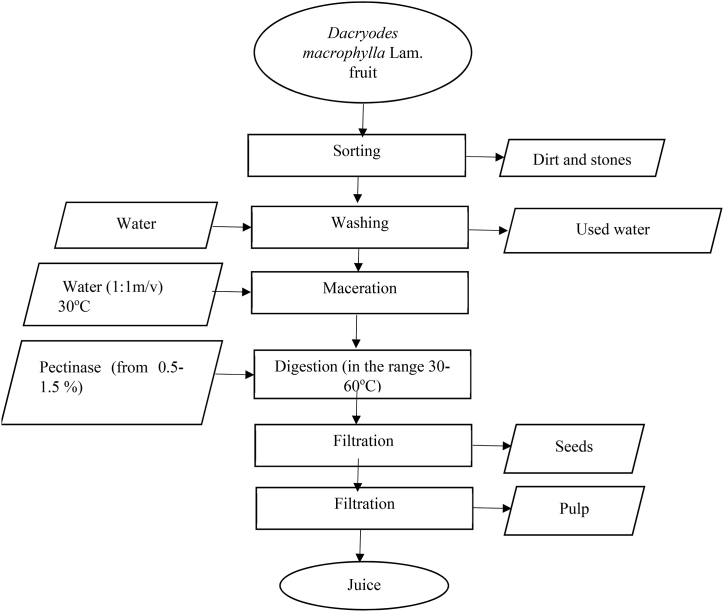
Block diagram representing enzyme-assisted juice extraction process from *Dacryodes macrophylla* Lam. fruit [18].

Juice from *Dacryodes macrophylla* fruits was extracted with the help of pectinase enzyme as described by Ref. [18] and illustrated in Fig. 1. Pectinase was added to a known volume of the macerated fruits and left to digest in the range of 30–60 min before filtering. After the digestion period, it was then filtered through a clean sterile muslin cloth to get clear juice free from seeds and other unwanted particles.

### 2.3 Experimental design

A Central Composite Design (CCD) was used, where 100 ml of homogenized pulp was treated with the enzyme. The independent parameters, such as enzyme concentration X_1_ (0.5–1.5v/v %), incubation temperature, X_2_ (20–60 °C), and incubation time, X_3_ (30–60 min), were considered to generate the experimental matrix consisting of 16 runs as shown on Table 1.

**Table 1:** Experimental matrix in real values for the optimization of enzyme-assisted juice extraction.

Experimental runs	REAL AND CODED VALUES		
	X_1_ Enzyme concentration (%)	X_2_ Incubation temperature (°C)	X_3_ Incubation time (minutes)
1	1 (0)	73.6 (1.682)	45 (0)
2	1 (0)	40 (0)	45 (0)
3	0.5 (-1)	60 (1)	60 (1)
4	0.5 (-1)	60 (1)	30 (-1)
5	0.5 (-1)	20 (-1)	60 (1)
6	0.2 (-1.682)	40 (0)	45 (0)
7	1.5 (1)	20 (-1)	60 (1)
8	0.5 (-1)	20 (-1)	30 (-1)
9	1.5 (1)	60 (1)	30 (-1)
10	1 (0)	40 (0)	45 (0)
11	1.8 (1.682)	40 (0)	45 (0)
12	1.5 (1)	60 (1)	60 (1)
13	1 (0)	40 (0)	70.23 (1.682)
14	1.5 (1)	20 (-1)	30 (-1)
15	1 (0)	6.36 (-1.682)	45 (0)
16	1 (0)	40 (0)	19.7 (-1.682)

After the specified treatment, the suspension was kept at −2 °C for 5 min for the inactivation of the enzyme adapted from cold denaturation of enzymes as discussed by Ref. [19]. The filtrate was measured, yield calculated, and further analyzed for different physicochemical properties (TSS, pH, and Vitamin C). The effects of variables in terms of linear, quadratic, and interaction terms can be explained by a mathematical model by Ref. [20] as shown below:

(1) $$Y=\beta_{0}+\beta_{1}X_{1}+\beta_{2}X_{2}+\beta_{3}X_{3}+\beta_{12}X_{1}\;X_{2}+\beta_{13}X_{1}\;X_{3}+\beta_{23}X_{2}\;X_{3}+\beta_{11}X_{1}^{2}+\beta_{22}X_{2}^{2}+\beta_{33}X_{3}^{2}+E$$

where Y is the experimental responses; X_1_, X_2_, and X_3_ are the experimental variables; β_0_ is the intercept; β_1_ to β_3_ are linear coefficients; β_11_ is the quadratic term, and β_12_ is the coefficient of the interaction terms.

### 2.4 Fermentation of juice extracted from *Dacryodes macrophylla* lam. Fruit

**Fig. 2:**
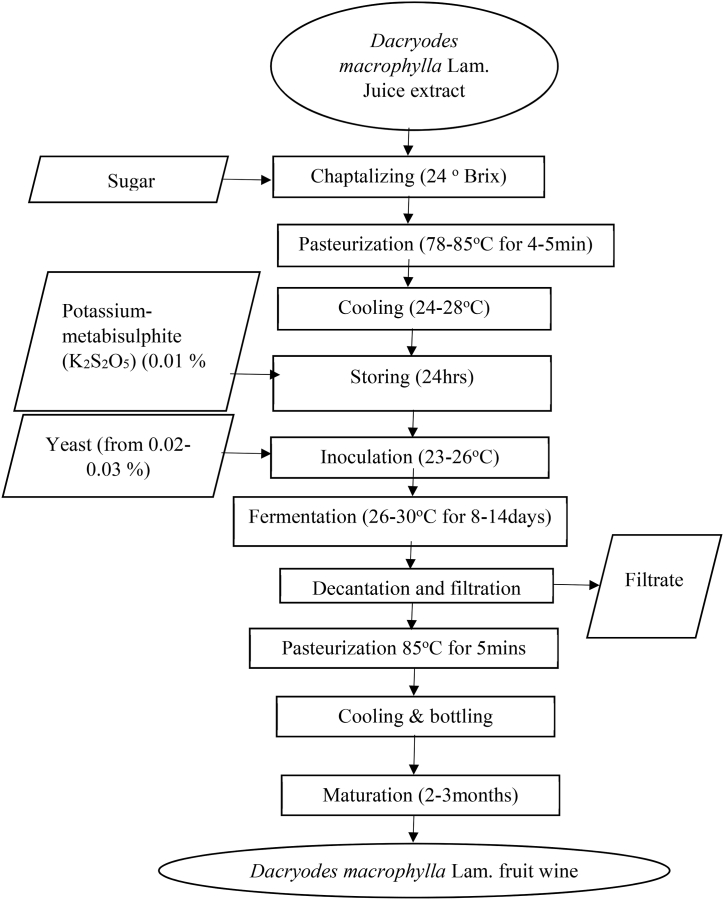
Block diagram for processing of wine from *Dacryodes macrophylla* Lam. juice extract [21].

The fermentation of the juice extracted from *Dacryodes macrophylla* Lam. fruit was carried out as described by Ref. [21] and illustrated in Fig. 2. The juice was chaptalized to 24^0^Brix and pasteurized at 80 ֯C for 5min. It was then cooled to 28 ֯C using a water bath and 0.01% of potassium metabisulphite was added to the must and kept for 24 h. The must was inoculated for 14 days with 0.03% yeast which was previously activated for 10 min s in warm water. Titratable acidity (TA), pH, and total soluble solids (TSS) were monitored every two days during the fermentation. After the fermentation period, it was then filtered, pasteurized at 85 ֯C 5min, and transferred to a sterile bucket for maturation for 2–3 months to improve the clarity and flavor of the wine.

### 2.5 Analytical methods

#### 2.5.1 Juice yield from *Dacryodes macrophylla* lam. Fruit

Juice yield was calculated as the volume of clear juice obtained from 100 ml of macerated pulp with seeds. The treated pulp was passed through a muslin cloth [22]. The percent juice content of the atom was given as.

$$\%\;Juice\;\left(v/v\right)=\frac{volume\;of\;filtrate\left(ml\right)}{volume\;of\;macerated\;fruit\;\left(ml\right)}*100$$

#### 2.5.2 Total soluble solids TSS/sugar content

The sugar content was measured using a refractometer (Fisher brand Analog Brix Refractometer, Model 12-561-338). This was done by placing a small amount of sample on a clean dry prism surface of the refractometer. The value of sugar was read and recorded in ^0^Brix (I·S·O 13815, 1993).

#### 2.5.3 Titratable acidity

The titratable acidity was described by Ref. [23]**.**

#### 2.5.4 pH

The pH of samples was measured using a hand-held pH meter (pHep HANNA instrument, Singapore). About 20 ml of the sample was put in a cup. The electrode of the pH meter was then dipped into the sample and the pH was read from the screen and recorded.

#### 2.5.5 Determination of vitamin C (ascorbic acid) content

Vitamin C content was estimated by the method described by Ref. [22].

5 ml of the juice was taken into a 25 ml volumetric flask and the volume was completed to the mark with 5% metaphosphoric acid-10% acetic acid solution. 5 ml of the solution was taken into another 25 ml volumetric flask. Few drops of bromine water were added to oxidize the ascorbic acid to dehydroascorbic acid. Then a few drops of thiourea were added to remove the excess bromine and thus a clear solution was obtained. One mL of glacial acetic acid and 1 ml of 2, 4- DNPH was added to the sample solution. For the completion of the reaction, the samples were kept at 37 ֯C for 3 h in a thermostatic water bath (B. Bran Scientific and Instrument Company, England).

After this incubation, the sample solutions were cooled in an ice bath for half an hour and treated with 5 ml of 85% H_2_SO_4_ with constant stirring. The volume was completed to the mark with distilled water. The absorbance of the sample was read at 521 nm using a UV–Visible spectrophotometer (UV 752(D), PEC Medical, USA).

#### 2.5.6 Alcohol content

The alcohol content was estimated using [24] alcohol equation

%Alcohol=(Si−Sf)*0.592

Si = Sugar content before fermentation (^0^Brix).

Sf = Final sugar content after fermentation (^0^Brix).

### 2.6 Sensory analysis

Sensory evaluation was done using a 30-man panel using the 9-point hedonic scale. The consent of all participants was obtained before the testing exercise as the only requirement for sensory evaluation in Cameroon since ethical Clarence is not a pre-requite for sensory evaluation. A coded sample was presented to 30 panellists who were familiar with wine consumption for the sensory evaluation. The panellist was then briefed on how the sensory evaluation was to be carried out especially as they were dealing just with a single sample. Sensory analysis on color, clarity, mouthfeel, flavor, after taste, and overall acceptability was done on the wine prepared from *Dacryodes macrophylla* fruit juice using the questionnaire below. The 9-point hedonic scale ranged from extremely dislike to extremely like as indicated below.

A. Personal information
B. Evaluation of wine

*Rate the wine sample with a number between 1 and 9 based on the scale below*:

After tasting each sample, rinse your mouth with clean water and wait for at least 30 s before moving to the next sample.

### 2.7 Microbial analysis

This aspect of analysis was based on the assessment of the possible microbes present in the wine samples as discussed by Ref. [25].

### 2.8 Data analysis

Experiments were carried out in triplicates and data obtained was subjected to analysis of variance (ANOVA) and Duncan test to assess the effect of different factors on the response and the differences between means respectively using STATGRAPHICS centurion version XVII. The p-value ≤0.05 was used for significant effect or difference, *R*^2^ value > 0.7, and/or standard error < 10% was used for model validation [20]. The graphs were plotted using sigma plot 14.5, Statgraphics, and excel software.

## 3 Results and discussion

### 3.1 Effects of extraction process parameters on response

Following the experiments and analysis, the obtained results for the responses; Y_1_ = Juice yield (%); Y_2_ = Total soluble solids TSS (^0^Brix); Y_3_ = pH; Y_4_= Vitamin C (mg/g) were as presented in Table 2 while Table 3 presents the modelling of enzyme assisted juice extraction process.

**Table 2:** Central composite design and response results for *Dacryodes macrophylla* juice.

Run	Experimental factors			Responses			
	×1	×2	×3	Y1	Y2	Y3	Y4
1	1.0	73.6	45	70 ± 1.41	10.4 ± 0.28	3.35 ± 0.03	11.32 ± 0.13
2	1.0	40	45	69 ± 1.41	9.9 ± 0.71	3.25 ± 0.01	12.15 ± 0.09
3	0.5	60	60	72 ± 1.41	10.4 ± 0.14	3.32 ± 0.03	11.93 ± 0.56
4	0.5	60	30	71 ± 0.0	10.4 ± 0.0	3.27 ± 0.04	11.53 ± 0.09
5	0.5	20	60	60 ± 2.83	9.8 ± 0.42	3.29 ± 0.01	15.32 ± 0.26
6	0.2	40	45	76 ± 1.41	10.5 ± 0.0	3.30 ± 0.0	13.67 ± 0.09
7	1.5	20	60	70 ± 0.0	10.4 ± 0.28	3.25 ± 0.01	15.50 ± 0.17
8	0.5	20	30	62 ± 1.41	10.0 ± 0.28	3.25 ± 0.04	15.56 ± 0.09
9	1.5	60	30	74 ± 2.83	10.4 ± 0.42	3.35 ± 0.0	12.39 ± 0.09
10	1.0	40	45	76 ± 1.41	10.5 ± 0.14	3.31 ± 0.03	13.73 ± 0.17
11	1.8	40	45	80 ± 1.41	10.6 ± 0.14	3.39 ± 0.01	14.04 ± 0.09
12	1.5	60	60	81 ± 0.0	10.6 ± 0.0	3.29 ± 0.01	12.23 ± 0.13
13	1.0	40	70.2	70 ± 1.41	10.4 ± 0.14	3.29 ± 0.0	12.81 ± 0.17
14	1.5	20	30	58 ± 2.83	9.7 ± 0.57	3.26 ± 0.03	14.61 ± 0.13
15	1.0	6.36	45	59 ± 0	9.8 ± 0.28	3.25 ± 0.04	14.21 ± 0.09
16	1.0	40	19.7	68 ± 2.83	9.9 ± 0.0	3.25 ± 0.01	13.73 ± 0.09

X_1_ = Enzyme concentration (%), X_2_ = Incubation temperature (֯C), X_3_ = Incubation time (mins); Y_1_ = Juice yield (%); Y_2_ = Total soluble solids TSS (^0^Brix); Y_3_ = pH; Y_4_= Vitamin C (mg/g).

**Table 3:** Modelling of the enzyme-assisted juice extraction process.

constant	Y1		Y2		Y3		Y4	
	Const	p-value	Const.	p-value	Const.	p-value	Const.	p-value
β_0_	16.5797		8.76		3.18		16.580	
β_1_	−2.26574	0.0923	- 0.865	0.3268	- 0.17	0.2831	- 2.27	0.6219
β_2_	−0.04069	**0.0024**	0.04	**0.0068**	- 0.002	**0.0346**	- 0.04	**0.0007**
β_3_	−0.01195	0.1398	0.03	0.0735	0.007	0.2724	- 0.01	0.9450
β_12_	0.014126	0.9633	- 0.004	0.5559	0.0015	0.2233	0.01	0.6187
β_13_	−0.00486	0.2661	0.014	0.1903	- 0.0019	0.2313	- 0.01	0.8949
β_23_	−0.00044	0.5228	- 0.0002	0.4128	- 0.0000028	0.9448	−0.0004	0.6505
β_11_	1.07309	0.7109	0.274	0.2990	0.106	**0.0272**	1.07	0.2829
β_22_	−0.00022	**0.0127**	- 0.0002	0.2551	0.000023	0.3996	−0.0002	0.7371
β_33_	0.00037	0.0705	- 0.0003	0.3363	- 0.000044	0.3633	0.0004	0.7423
R^2^	0.91		0.83		0.76		0.86	
E	4.11318		0.2193		0.0343131		0.828727	

Y_1_ = % Yield, Y_2_ = TSS, Y_3_ = pH, Y_4_= Vitamin C, Values in bold signifies (p˂0.05).

The models were considered valid as the *R*^2^ value for each response was >0.7 and the Standard Error of Est < 10% [21]. Thus, sufficiently explains the effect of enzyme concentration, incubation temperature, and incubation time on the % yield, TSS, pH, and Vitamin C content of enzymatically extracted juice of atom fruit.

The model equation obtained from the regression coefficient for the factors were;

(2) Juice Yield (Y1) = 40.22 + 0.09 × 3 - 0.009 × 22–0.003 × 2 × 3 - 0.011 × 32

(3) TSS(Y_2_) = 8.76 + 0.04 × _2_

(4) pH(Y_3_) = 3.18–0.002 × _2_ + 0.106 × _1_^2^

(5) Vitamin C (Y_4_) = 16.5797–0.04 × _2_

#### 3.1.1 Effect of enzyme concentration, incubation temperature, and time on the juice yield of *Dacryodes macrophylla* fruit

**Fig. 3:**
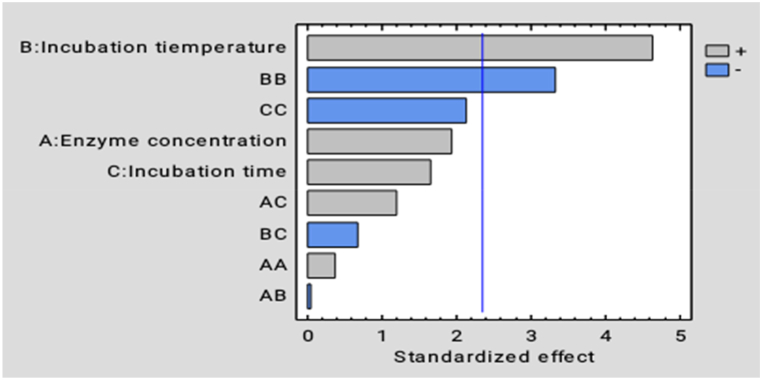
Effects of different process factors and their interactions on juice yield of *Dacryodes macrophylla* fruit. Blue vertical line stands for Significant difference at p˂ 0.05, horizanta bars which touch or crossed the line thus shows a significant difference. (For interpretation of the references to color in this figure legend, the reader is referred to the Web version of this article.)

The effect of the process parameters on the juice extraction from *Dacryodes macrophylla* fruit is shown in Fig. 3. From the analysis of variance as shown in Table 3 and the Pareto in Fig. 3, the incubation temperature (X_2_) and incubation temperature-squared (X_2_^2^) significantly affect the juice yield at (p˂ 0.05). However, enzyme concentration, incubation time, and other interactions did not show any significant effect on the juice yield.

**Fig. 4:**
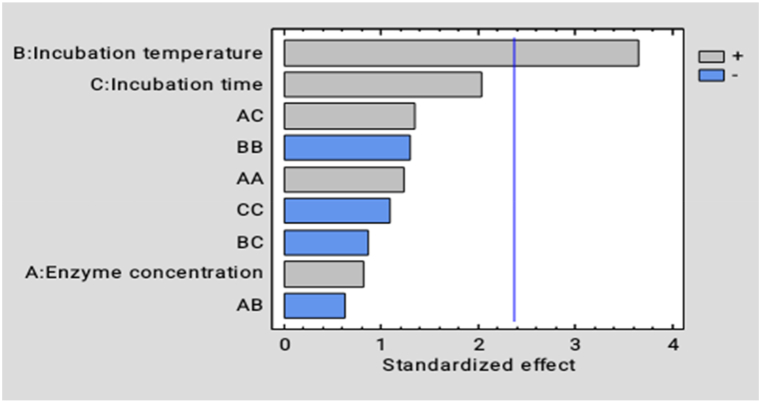
Effects of different process factors and their interactions on the total soluble solid content of *Dacryodes macrophylla* juice. Blue vertical line stands for Significant difference at p˂ 0.05, horizanta bars which touch or crossed the line thus shows a significant difference. (For interpretation of the references to color in this figure legend, the reader is referred to the Web version of this article.)

**Fig. 5:**
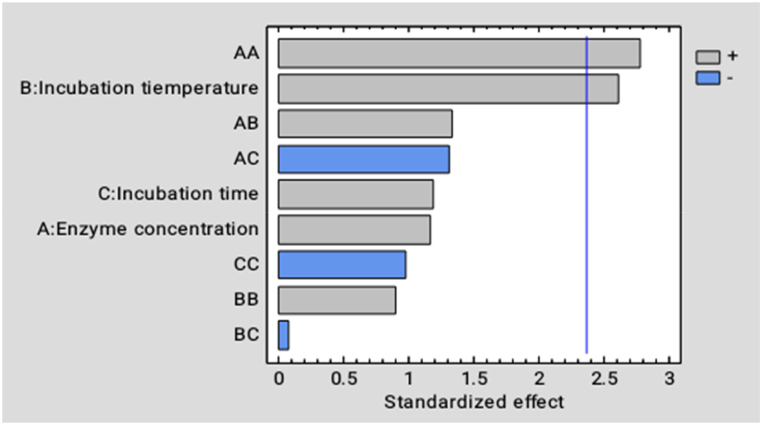
Effects of different factors and their interactions on pH of *Dacryodes macrophylla* fruit juice. Blue vertical line stands for Significant difference at p˂ 0.05, horizanta bars which touch or crossed the line thus shows a significant difference. (For interpretation of the references to color in this figure legend, the reader is referred to the Web version of this article.)

**Fig. 6:**
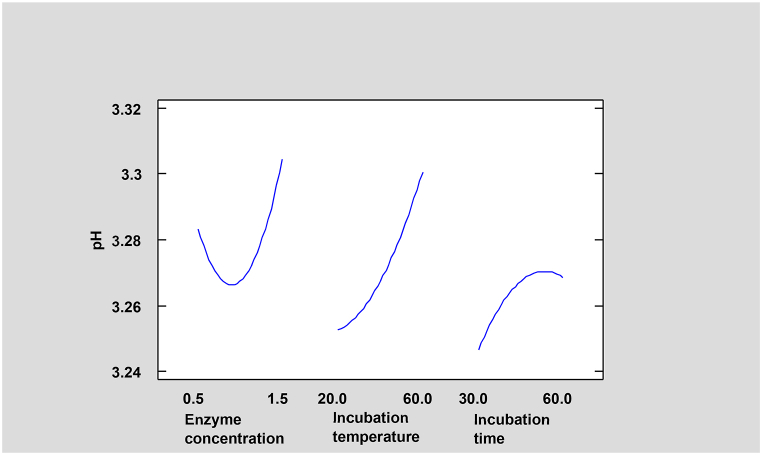
Contour plots of an estimated response surface for pH of *Dacryodes macrophylla* fruit juice in response to varying enzyme concentration, incubation temperature and time.

**Fig. 7:**
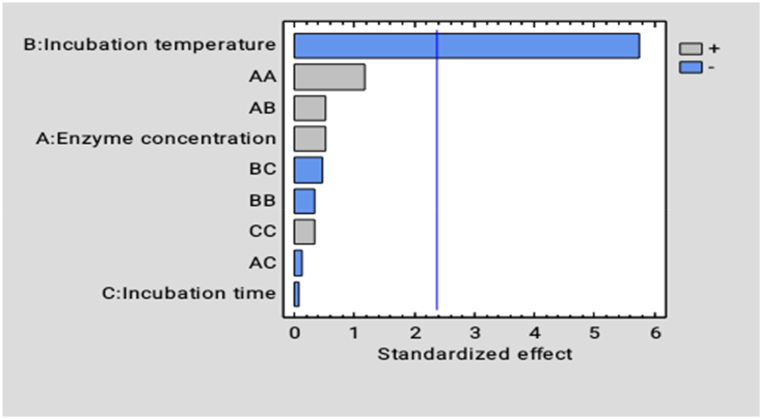
Effects of different factors and their interactions on ascorbic acid (vitamin c) content of *Dacryodes macrophylla* fruit juice. Blue vertical line stands for Significant difference at p˂ 0.05, horizanta bars which touch or crossed the line thus shows a significant difference. (For interpretation of the references to color in this figure legend, the reader is referred to the Web version of this article.)

Incubation temperature (X_2_) has a positive significant effect (p < 0.05) while incubation temperature-squared (X_2_^2^) has a negative significant effect (P < 0.05) on juice yield. However, enzyme concentration (X_1_), enzyme concentration -squared (X_1_^2^), incubation time (X_3_), and the interaction between enzyme concentration-incubation time (X_1_X_3_) have positive significant (p˃ 0.05) effects on Juice yield. Also, the interactions; enzyme concentration-incubation temperature (X_1_X_2_), incubation temperature-incubation time (X_2_X_3_), and the quadratic incubation time (X_3_^2^) have a negative insignificant effect on the juice yield (Fig. 7).

A significant increase in juice yield with an increase in incubation temperature (X_2_) and enzyme concentration (X_1_) is attributed to the fact that an increase in enzyme concentration and incubation time will also increase the rate of the enzyme action as it degrades intracellular walls and pectin thereby releasing more free water into the system thus resulting to greater juice yield [26]. Incubation time (X_3_) also increased juice yield due to a longer time of exposure for enzyme action to completely take place.

A significant decrease in juice yield with quadratic effects of incubation temperature (X_2_^2^) and incubation time (X_3_^2^) could be attributed to the fact that at higher temperatures the enzyme is denatured as they are protein in nature, thus their action in releasing trapped juice in cell walls is stopped leading to lower juice yield [27].

Also, an increase in incubation time-incubation temperatures (X_2_X_3_) combine effect decreases juice probably because at higher temperatures where enzyme has been denatured no matter how long you keep the reaction process, they won't be any increase in juice whereas at these high temperatures some of the water in juice may start evaporating resulting in a lower juice yield [28]. However, enzyme concentration-squared (X_1_^2^) will yield a greater juice yield as the more the enzyme higher the rate of reaction [16].

#### 3.1.2 Effect of enzyme concentration, incubation temperature, and incubation time on the total soluble solids (TSS) of *Dacryodes macrophylla* fruit

From the ANOVA analysis for total soluble solids, incubation temperature (X_2_) was found to significantly (P˂0.05) affect the total soluble solids content of *Dacryodes macrophylla* fruit juice. Whereas all the other factors squared terms and interaction terms had no significant effect on the total solid content of atom juice as shown in Fig. 4.

Incubation temperature (X_2_) has a positive significant (p˂0.05) effect on the total soluble solids content whereas incubation time (X_3_), enzyme concentration (X_1_), enzyme concentration-squared (X_1_^2^), and the interaction term; enzyme concentration-incubation time (X_1_X_3_) all have a positive insignificant effect (p˃0.05) on the total soluble solids. However, incubation temperature-squared (X_2_^2^), incubation time squared (X_3_^2^), and the interactions; incubation temperature-incubation time (X_2_X_3_), enzyme concentration-incubation temperature (X_1_X_2_) were found to have an insignificant negative effect on the total soluble solid content of the samples of atom juice.

The Total soluble solid content was observed to increase with an increase in incubation temperature (×2), enzyme concentration (×1), and incubation time (×3). This could be attributed to the fact that high optimal temperatures favoring enzyme activity and at a higher enzyme concentration for a long incubation time leads to a greater degree of tissue breakdown, releasing more compounds such as sugars [29], The negative effect of incubation temperature-squared (×22), incubation time squared (×32), and the interactions; incubation temperature-incubation time (X2X3), enzyme concentration-incubation temperature (X1X2) on the total soluble solid content could also be attributed to the fact that at very high temperatures enzyme denaturation occurs thus no more breakdown of tissues to release soluble solids and also incubation at a longer time may result to the onset of fermentation which will instead use up some of the soluble solids resulting to lower total soluble solids at the end.

#### 3.1.3 Effect of enzyme concentration, incubation temperature, and incubation time on the pH of *Dacryodes macrophylla* fruit

From the ANOVA, enzyme concentration-squared (X_1_^2^) and incubation temperature (X_2_) were found to significantly affect pH (p˂0.05). Whereas enzyme concentration (X_1_), incubation time (X_3_), incubation temperature-squared, and the interaction; enzyme concentration-incubation temperature (X_1_X_2_) had a positive insignificant effect (p <0.05) on the pH of atom juice as shown on Fig. 5.

Incubation time-squared (X_3_^2^) with the interactions; enzyme concentration-incubation time (X_1_X_3_) and incubation temperature-incubation time (X_2_X_3_) had a negative insignificant effect on the pH of *Dacryodes macrophylla* fruit juice.

The contours of an estimated response surface for pH as seen in Fig. 6 indicate an increase in pH values with an increase in enzyme concentration (X_1_) which differs from the works of [29] whose pH decreased with an increase in enzyme concentration. According to Ref. [30], a decrease in pH from 4.5 to 3.0 could increase the shelf life of juice to about 3 times.

#### 3.1.4 Effect of enzyme concentration, incubation temperature, and incubation time on the vitamin C content of enzymatically extracted juice of *Dacryodes macrophylla* fruit

From the ANOVA, incubation temperature (X_2_) had a significant effect (p <0.05) on the Vitamin C content of atom juice, whereas all other factors had no significant effect on the Vitamin C content of atom juice as seen in Fig. 7.

Incubation temperature (X_2_), incubation time (X_3_), incubation temperature-incubation time (X_2_X_3_), enzyme-incubation time (X_1_X_3_), and incubation temperature-squared (X_2_^2^) were all seen to have a negative effect on vitamin c content of atom juice. Also, enzyme concentration (X_1_), enzyme concentration-squared (X_1_^2^), incubation time-squared (X_3_^2^), and the interaction term enzyme concentration-incubation temperature all show a positive effect on the vitamin c content of atom juice.

Conclusively, results of multiple response optimization analysis revealed optimized conditions for enzyme concentration, incubation temperature, incubation time to be 1.84%, 49.02 ֯C and 43.58 min s respectively with optimum response desirability of 79.47%, 10.61^0^Brix, pH of 3.39, 15.56 mg/ml Vit C concentration and titratable acidity value of 0.35.

### 3.2 Effect of enzymatic treatment on juice yield and physicochemical properties of *Dacryodes macrophylla* juice

The juice yield and physicochemical properties of the extracted juice were as shown in Table 4.

**Table 4:** Physicochemical parameters of extracted juice from *Dacryodes macrophylla* fruit.

	Physicochemical Parameters				
	% Juice yield	TSS (^0^Brix)	pH	Vitamin C (mg/ml)	Titratable acidity
Enzyme Treated	81 ± 0.28^a^	10.6 ± 0.14^a^	3.39 ± 0.03^a^	15.56 ± 0.03^a^	0.35 ± 0.04^a^
Untreated	46 ± 0.28^b^	9.5 ± 0.28^b^	3.25 ± 0.07^b^	15.71 ± 0.037^a^	0.35 ± 0.03^a^

Values are Mean ± standard deviation with column scores with superscript (a and b) being significantly different at p < 0.05.

Enzyme treatment had a great impact on the % juice yield of samples as it was as high as 81 ± 0.70% for the treated samples whereas that of the untreated sample was 46 ± 0.70%. Generally, the juice yield (58–81%) was similar to those reported by Refs. [27,31,32]. Had juice yields of 84.24%, 88.53%, and 81% respectively. This can be attributed to the fact that the enzyme breakdown tissue cell walls realizing water trapped within them thus increasing juice yield.

Enzymatic extraction also increased the TSS of juice from various fruits. The TSS content of the enzyme-treated sample had a value of 10.6 ± 0.2^0^Brix which was significantly higher than the untreated sample with a value of 9.5 ± 0.21 ^0^Brix. These results were in line with the works of [33,34] who also reported an increase in the TSS content on their enzyme-treated samples. This could be attributed to the fact that enzymes help in tissue breakdown, releasing more compounds such as sugars [29].

The pH of atom juice initially decreased with an increased in enzyme concentration below 1% which is in line with the works carried out by Ref. [29] but then significantly increased at enzyme concentrations above 1%. The enzyme-treated samples had a pH reading of 3.39 ± 0.01 greater than that of the untreated sample 3.25 ± 0.01.

The Vitamin C content of enzyme-extracted juice decreased to 15.56 ± 0.2 mg/ml sample as compared to that of the untreated 15.71 ± 0.04 mg/ml, which could be due to the oxidation of ascorbic acid during the enzyme-assisted juice extraction process and also due to its degradation due to processing factors as increase temperature (Sharma et al., 2014). The enzyme treatment did also seem to increase the ascorbic acid content significantly at enzyme concentrations above 1% [29]. found in apple pomace that the remaining ascorbic acid content was unaffected by the increase in enzyme concentration.

[33] found that the titratable acidity for enzymatically extracted juice from guava puree increased which differed from this work as the enzyme-treated samples' titratable acidity tend to be the same and slightly decreased for some of the treated samples than that of the untreated sample. The increase in the latter was explained by the addition of citric acid during enzymatic extraction and liberation of galacturonic acid inducted by pectinase adjunction. The titratable acidity values for but treated and the untreated sample had no significant difference (P < 0.05) with both having a value of 0.35 ± 0.05.

### 3.3 Physico-chemical parameters of *Dacryodes macrophylla* fruit wine during fermentation

#### 3.3.1 Evolution of pH and titratable acidity during primary fermentation

The evolution of the pH and titratable acidity during primary fermentation of the extracted juice from *Dacryodes macrophylla* juice. To wine within 14 days was as presented in Table 5.

**Table 5:** Evolution of pH and titratable acidity during primary fermentation.

Days	Parameter	
	pH	Titratable acidity
0	3.42 ± 0.07^a^	0.16 ± 0.05^a^
2	3.40 ± 0.07^a^	0.27 ± 0.02^bc^
4	3.37 ± 0.07^b^	0.35 ± 0.04^c^
6	3.33 ± 0.07^c^	0.4 ± 0.02^cd^
8	3.31 ± 0.07^cd^	0.45 ± 0.02^d^
10	3.29 ± 0.07^de^	0.50 ± 0.02^d^
12	3.28 ± 0.07^ef^	0.52 ± 0.00^d^
14	3.26 ± 0.07^fg^	0.51 ± 0.00^d^

Column scores with subscript (a, b, c, d, bc, cd, de, ef, fg) are significantly different at (P < 0.05).

There was a decrease in pH with an increase in days of fermentation and starting from an initial value of 3.42 on day zero to a final value of 3.26 on day 14. Theoretically, sugars are converted to alcohols, then alcohols to aldehydes, aldehydes to ketones, and ketones are finally converted to acids during fermentation [35]. The pH of the wines lay within the favorable pH range for red wines 3.3 to 3.6 [36]. The general drop in pH as fermentation evolved follows the same trend as results reported by Ref. [14]. for the production of mixed fruit wines using yeast isolated from palm wine. The sharp changes (p < 0.05) in pH after day one could be a result of the rapid fermentation rate due to the availability of excess sugar and favorable conditions for yeast metabolism releasing end products.

Titratable acidity increased during fermentation; the titratable acidity was seen to rise from a minimum of 0.16 ± 0.05 to 0.52 ± 0.02 which was in line with the increase in titratable acidity for roselle wine specified by Ref. [37]. An increase in titratable acidity was a result of the utilization of nutrients by yeast and the production of organic acids during fermentation [38]. This observation is similar to work done on roselle wine in which the TA increased from 0.69 to 0.75% at the end of 30 days of aging after fermentation [42].

#### 3.3.2 Alcoholic content

The alcoholic content was at 10.24 ± 0.2 which is within the range for still table wine as stated by EAS, (2017) (range from 6.5 to 16.5%).

#### 3.3.3 Microbial analysis

Table 6 shows the means ± SD of triplicate determinants for colony forming units per ml of wine samples for total bacteria count (TBC), total coliform count (TCC), and yeast and mold enumeration.

**Table 6:** Results of microbial count in *Dacryodes macrophylla* juice wine.

SAMPLE	TBC	TCC	Yeast and mold
	(10^2^ cfu/ml)	(10^1^ cfu/ml)	(10 ^2^ cfu/ml)
values	0.5 ± 0.1	0	0.4 ± 0.3
Norms	1	0	1

Table 6 shows the mean ± SD of the colony forming units per ml (CFU/ml) of TBC, TCC, and the number of yeast and mold found in the prepared wine sample. The sample showed 0 colony forming units per ml of the total coliform count, and 0.5 ± 0.1 CFU/ml of total bacteria count. However, the sample showed colony forming of yeast units per ml of wine which was 0.4 ± 0.2 CFU/ml. These were all below the limits as stated by EAS, 2017.

The under 1 × 10^2^ CFU/ml of total bacteria count and total coliform count were presumed to be a result of aseptic processing and packaging techniques and the use of potassium metabisulphite which prevented the growth of microbes. The presence of CFU/ml of yeast and mold in the wine sample was assumed to be because live yeast cells were used for wine fermentation which were not effectively killed during pasteurization at the end of fermentation or contamination during inoculation.

#### 3.3.4 Sensory analysis

Sensory evaluation was done on the wine prepared using fresh juice extract of *Dacryodes macrophylla* fruit as starting material. Table 7 shows the mean sensory scores for the wine. The sensory score of 7.23 ± 1.31, 7.27 ± 1.26, 7.73 ± 1.18, 7.00 ± 1.57, 6.63 ± 1.52, 7.17 ± 1.55, and 7.97 ± 1.17 for color, clarity, flavor, mouthfeel, alcoholic burn, after taste and overall acceptability respectively. From the sensory evaluation still, most of the panellists choose their overall acceptability based on the after taste of the wine, and also the overall acceptability reading was closest to like very much.

**Table 7:** Sensory evaluation results for wine from *Dacryodes macrophylla* fruit.

Sample	Color	Clarity	Flavor	Mouthfeel	Alcoholic burn	After taste	Overall acceptability
**values**	7.23 ± 1.31^a^	7.27 ± 1.26^a^	7.73 ± 1.18^a^	7.00 ± 1.57^a^	6.63 ± 1.52^a^	7.17 ± 1.55^a^	7.97 ± 1.17^a^

Values are Mean ± standard deviation (n = 9). (a); values with same superscript letters are not significantly different (P > 0.05).

## 4 Conclusion

This work aimed to use pectinase to extract juice from *Dacryodes macrophylla* fruit, ferment, and test for the acceptability of its wine. The results of this research showed that enzymes greatly increase the juice yield and total soluble solids of the atom fruit. The optimum processing condition in the extraction of juice from the atom fruit were 1.84% enzyme concentration, 49.02 °C Incubation temperature, and 43.58 min Incubation time. The wine produced from the extracted juice had physicochemical and microbial quality within the limits as stated by EAS, 2017. Also, the wine was generally accepted by all with a general acceptability score of 7.97. Thus, enzymes can be used to improve the juice yield of *Dacryodes macrophylla* fruit, and hence, the juice can be successfully valorised to a more shelf-stable product. The perspective of this research will aim at studying the effect of the production process and process parameter on the quality of wine produced from the extracted juice of *Dacryodes macrophylla.*

## Declaration of Interest

The authors declare that they have no known competing financial interests or personal relationships that could have appeared to influence the work reported in this paper
